# ATM Dependent DUSP6 Modulation of p53 Involved in Synergistic Targeting of MAPK and p53 Pathways with Trametinib and MDM2 Inhibitors in Cutaneous Melanoma

**DOI:** 10.3390/cancers11010003

**Published:** 2018-12-20

**Authors:** Chiao-En Wu, Tsin Shue Koay, Arman Esfandiari, Yi-Hsuan Ho, Penny Lovat, John Lunec

**Affiliations:** 1 Northern Institute for Cancer Research, School of Medicine, Newcastle University, Newcastle upon Tyne NE2 4HH, UK; jiaoen@gmail.com (C.-E.W.); T.S.Koay2@newcastle.ac.uk (T.S.K.); a.esfandiari@ucl.ac.uk (A.E.); yhho9196@gmail.com (Y.-H.H.); 2 Division of Hematology-Oncology, Department of Internal Medicine, Chang Gung Memorial Hospital at Linkou, Chang Gung University College of Medicine, Taoyuan 333, Taiwan; 3 Cancer Research UK Drug-DNA Interactions Research Group, UCL Cancer Institute, Paul O’Gorman Building, University College London, London WC1E 6BT, UK; 4 Dermatological Sciences, Institute of Cellular Medicine, Newcastle University, Newcastle upon Tyne NE2 4HH, UK; penny.lovat@newcastle.ac.uk

**Keywords:** trametinib, nutlin-3, RG7388, HDM201, p53, DUSP6, melanoma, MDM2, ATM

## Abstract

MAPK and p14^ARF^–MDM2–p53 pathways are critical in cutaneous melanomas. Here, synergistic combination of the MEK inhibitor, trametinib, with MDM2 inhibitors, nutlin-3/RG7388/HDM201, and the mechanistic basis of responses, for BRAF^V600E^ and p53^WT^ melanoma cells, are reported. The combination treatments induced higher levels of p53 target gene transcripts and protein products, resulting in increased cell cycle arrest and apoptosis compared with MDM2 inhibitors alone, suggesting trametinib synergized with MDM2 inhibitors via upregulation of p53-dependent pathways. In addition, DUSP6 phosphatase involvement was indicated by downregulation of its mRNA and protein following pERK reduction by trametinib. Furthermore, suppression of DUSP6 by siRNA, or inhibition with the small molecule inhibitor, BCI, at a dose without cytotoxicity, potentiated the effect of MDM2 inhibitors through increased ATM-dependent p53 phosphorylation, as demonstrated by complete reversal with the ATM inhibitor, KU55933. Trametinib synergizes with MDM2 inhibitors through a novel DUSP6 mechanism in BRAF^V600E^ and p53^WT^ melanoma cells, in which DUSP6 regulation of p53 phosphorylation is mediated by ATM. This provides a new therapeutic rationale for combination treatments involving activation of the ATM/p53 pathway and MAPK pathway inhibition.

## 1. Introduction

Alterations of genes involving both the mitogen-activated protein kinase (MAPK) (RAS-RAF-MEK-ERK) pathway and p14^ARF^–MDM2–p53 network are important in cutaneous melanoma [1,2,3]. The BRAF^V600E^ mutation occurs in 50–60% of melanomas [4,5], and small molecule kinase inhibitors targeting BRAF and MEK have become the standard treatment for patients with inoperable BRAF^V600E^ melanoma [6,7]. Despite impressive response rates in the early period of treatment, eventual relapse resulting from acquired resistance occurs for most patients. Therefore, additional therapeutic strategies are needed, and activating p53 function could be a parallel approach for melanoma treatment [8,9].

Mutations of p53 occur in approximately half of human cancers overall, but the frequency of p53 mutation varies among different cancer types. For melanomas, more than 80% are p53 wild-type (p53^WT^). However, loss of *CKDN2A*, which is a negative regulator of MDM2, is frequent in melanoma, and consequent increased MDM2 activity resulting in suppression of p53 function is therefore likely [4,10,11]. In the subgroup of BRAF mutated melanoma, ~90% of tumors are p53^WT^ [5]. Therefore, a therapeutic strategy of combining MAPK inhibitors and treatment reactivating p53 could have benefit in BRAF^V600E/^p53^WT^ melanoma. MDM2-p53 binding antagonists stabilize p53 by preventing MDM2-mediated inhibition and degradation of p53, which leads to activation of the p53 pathway in cancer cells retaining wild type p53, causing cell cycle arrest, apoptosis, and cell senescence [12]. Nutlin-3 was the first generation MDM2-p53 binding antagonist to be developed and shown to have efficacy in vitro and in vivo [13,14,15]. Both RG7388 (idasanutlin) [16] and HDM201 [17], orally bioavailable examples of the new generation of MDM2-p53 binding antagonists, efficiently suppress tumor growth in vivo, and clinical trials are currently ongoing to investigate the clinical efficacy of these and other MDM2 inhibitors in a range of cancers.

Dual specificity phosphatase 6 (DUSP6), *MAP kinase phosphatase-3* (MKP-3), a member of the DUSP family of phosphatases, dephosphorylates phospho-ERK (pERK), and has been reported to be regulated by p53 in HCT116 cells [18]. In addition, the MEK/ERK kinases have been reported to positively regulate *DUSP6* mRNA levels [19], and to phosphorylate and promote degradation of DUSP6 protein [20]. Our datamining shows that melanoma cell lines express the highest level of *DUSP6* mRNA among all cancer cell lines, according to the Cancer Cell Line Encyclopedia (CCLE) [21], and second highest among primary cancer tissues in cBioPortal (Supplementary Figure S1) [22]. Compared with BRAF^WT^ and NRAS^WT^ melanoma, significantly higher DUSP6 expression was found in melanoma cells with BRAF and NRAS mutations [23]. Furthermore, *DUSP6* expression was found to be a predictive biomarker for sensitivity to trametinib, and absence of *DUSP6* expression was associated with resistance to trametinib regardless of *RAF/RAS* status [24]. These collective observations indicate that DUSP6 plays an important autoregulatory role connecting the MAPK pathway and MDM2/p53 networks in cancer. We therefore hypothesized that DUSP6 is involved in the response to combination treatments targeting the MAPK and p53 pathways in BRAF^V600E^ melanoma.

In the current study, we tested combination treatments with a MEK inhibitor, trametinib, and MDM2 inhibitors, nutlin-3/RG7388/HDM201, in BRAF^V600E^/p53^WT^ melanoma cell lines, and explored the potential mechanism for the observed synergistic response to these combinations, revealing DUSP6 as a key connector for regulation between the MAPK and p53 pathways, and involvement of the ataxia telangiectasia (ATM) kinase.

## 2. Results

### 2.1. Combination Treatment of MDM2 Inhibitors and Trametinib in BRAF^V600E^ and p53^WT^ Melanoma Cells

**Figure 1:**
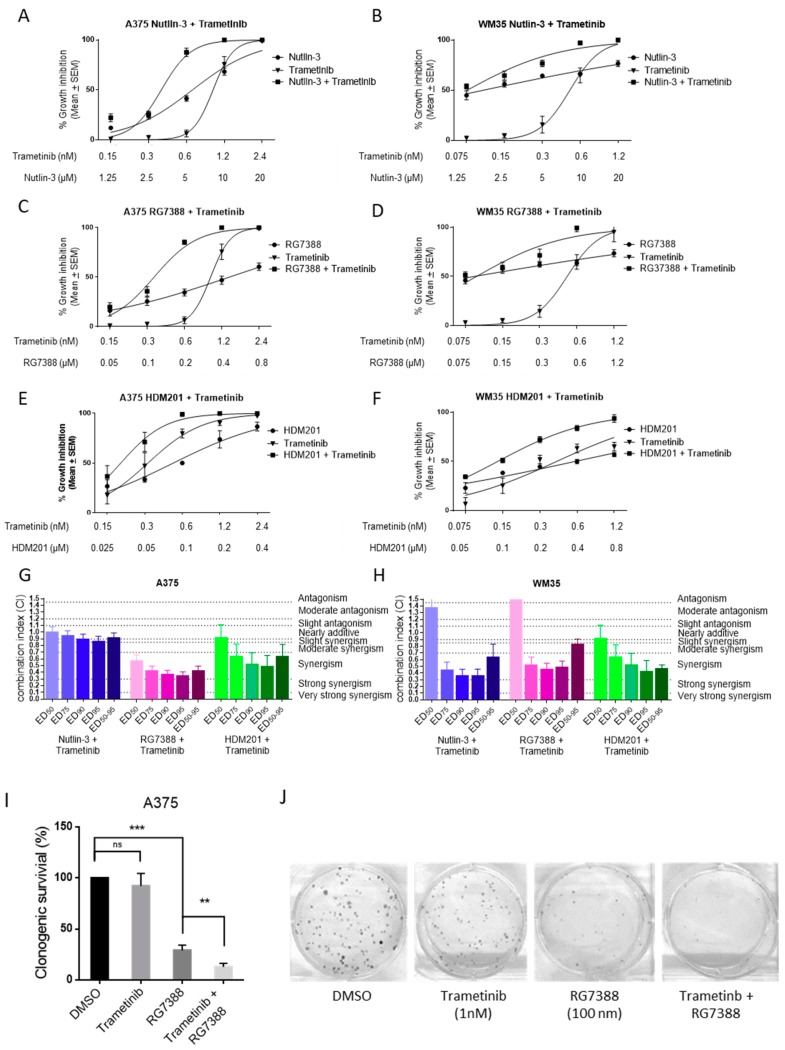
Synergistic effect of trametinib and MDM2 inhibitors on p53^WT^ and BRAF^V600E^ melanoma cells. Growth inhibition curves for trametinib, nutlin-3/RG7388/HDM201 alone or in combination at constant concentration ratios for 72 h with A375 (**A**,**C**,**E**) and WM35 (**B**,**D**,**F**). The CI values for nutlin-3/RG7388/HDM201 in combination with trametinib at ED_50_, ED_75_, ED_90_ and ED_50-95_ (average of CI values at ED_50_, ED_75_, ED_90_, and ED_95_) for A375 (**G**) and WM35 (**H**). (**I**,**J**) Clonogenic survival of A375 following RG7388 (100nM) and/or trametinib (1 nM) 72 h treatment. SEM, standard error of the mean. ns, *p ≥* 0.05; *, *p* < 0.05; **, *p* < 0.01; ***, *p* < 0.001.

The effect of MDM2 inhibitors, nutlin-3/RG7388/HDM201, in combination with trametinib, was investigated for BRAF^V600E^ and p53^WT^ melanoma cancer cell lines, A375 and WM35, using median-effect analysis [25]. A375 and WM35 were treated with trametinib, nutlin-3/RG7388/HDM201 alone, or in combination, at constant ratios, for 72 h (Figure 1A–F). To quantify and test the effect of combination treatment with MDM2 inhibitors and trametinib for synergy, the combination index (CI) was calculated using CalcuSyn, and almost all CIs for different combinations (except nutlin-3 and trametinib in A375) showed the combinations to be synergistic (CI < 0.9) at effective dose (ED)_50-95_, the average of ED_50_, ED_75_, ED_90_, and ED_95_ (Figure 1G,H). The degree of synergy varies with compound, dose effect level, and cell line. Generally, the CIs at ED_75_, ED_90_, and ED_95_ showed stronger synergistic effects than the CIs at ED_50_. Also, there was a favorable dose reduction index (DRI > 1.0), indicating the combination treatment could reduce the dose of each agent to achieve the same effects on the cells as the individual agents used singly (Table 1).

Clonogenic survival of A375 cells showed 1 nM trametinib produced no significant reduction in colony formation as a single agent, but significantly decreased the number of colonies when combined with RG7388 and HDM201, showing that trametinib enhanced the cytotoxic activity of MDM2 inhibitors (Figure 1I,J, Supplementary Figure S2).

### 2.2. Combination of Trametinib and MDM2 Inhibitors Increased the Induction of p53 Transcriptional Targets, Resulting in Further Cell Cycle Arrest and Apoptosis

**Figure 2:**
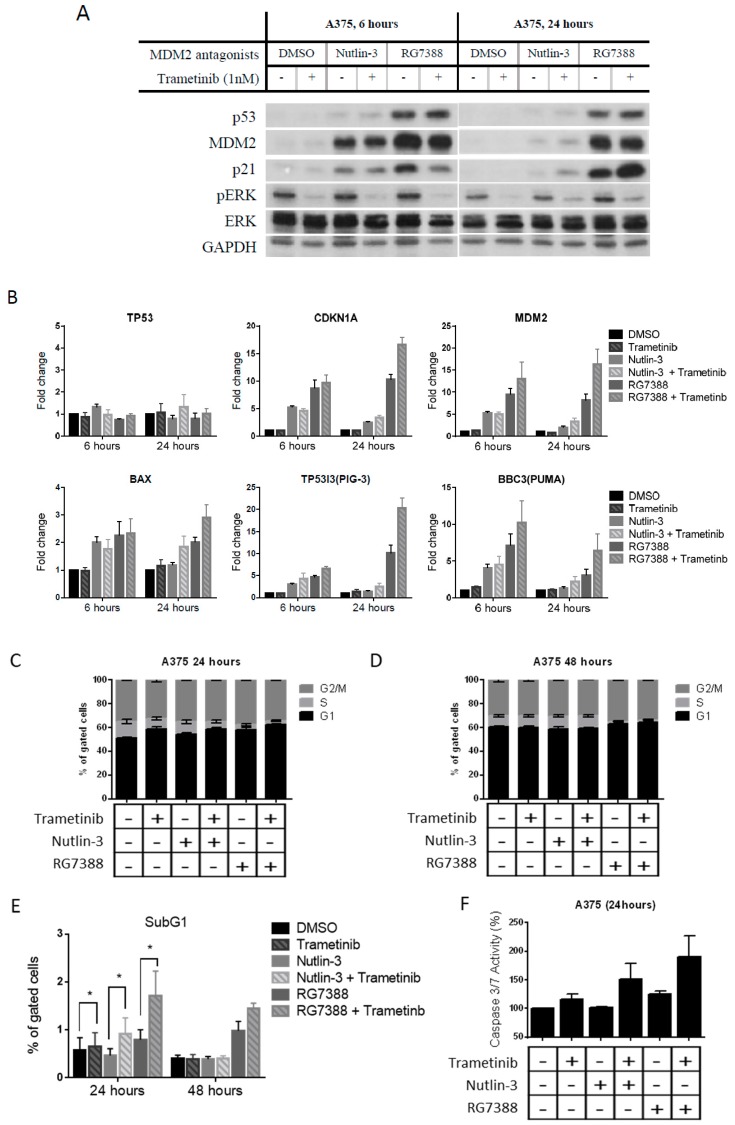
Trametinib and MDM2 inhibitor combination induction of p53 transcriptional targets resulting in enhanced cell cycle arrest and apoptosis of A375 cells. Analysis of A375 cells treated with nutlin-3 (1 µM)/RG7388(0.2 µM) ± trametinib (1 nM) for the indicated times, by immunoblotting (**A**), qRT-PCR (**B**), for cell cycle distribution changes (**C**,**D**), FACS sub-G1 signals (**E**), caspase 3/7 activity (**F**). *, *p* < 0.05.

**Figure 3:**
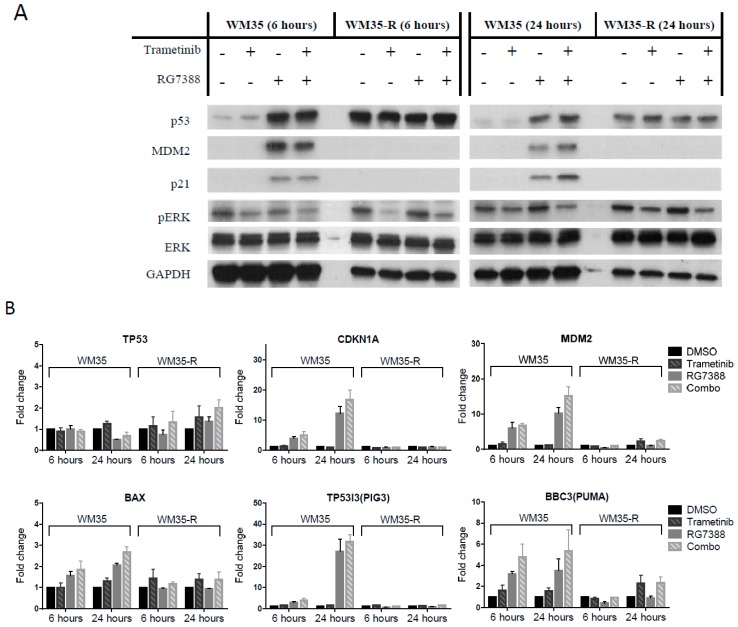

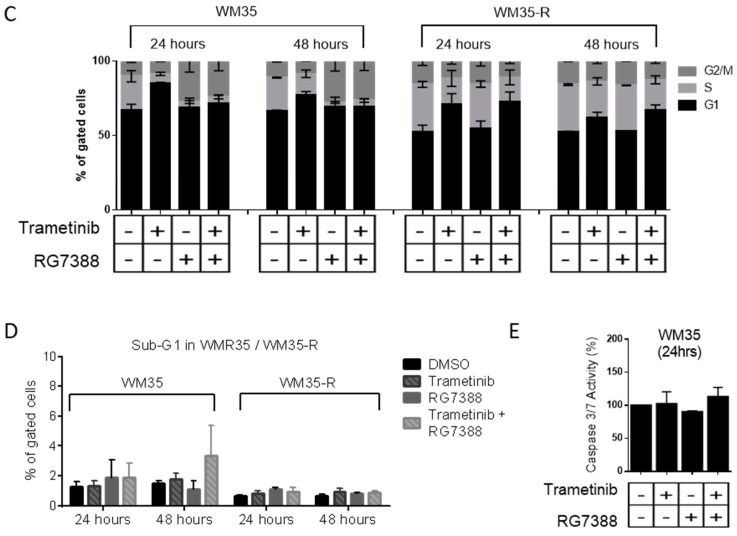
Trametinib and MDM2 inhibitor combination treatments in paired WM35 (p53^WT^)/WM35-R (p53^MUT^). Cells treated with RG7388 (0.2 µM) ± trametinib (1 nM) for indicated times and analyzed by immunoblotting (**A**) and qRT-PCR (**B**), by FACS analysis for cell cycle distribution changes (**C**) and sub-G1 events (**D**), and for caspase 3/7 activity (**E**). hrs, hours.

To understand the possible mechanisms for the synergistic effect of these combinations, immunoblotting and qRT-PCR was performed for A375 treated with trametinib (1 nM) ± nutlin-3 (1 µM)/RG7388 (0.2 µM) (Figure 2A,B) and also for WM35/WM35-R treated with trametinib (1 nM) ± RG7388 (0.2 µM) for 6 and 24 h (Figure 3A,B). The on-target effects of p53 stabilization and pERK suppression were observed after treatment with MDM2 inhibitors and trametinib, respectively, in A375 and WM35. Interestingly, after 24-h treatment, significantly higher p21 expression was found with combination treatment than for MDM2 inhibitors alone, without a readily observable increase of p53 protein. Expression of mRNA for the p53 transcriptional targets, *CDKN1A, MDM2, BAX, TP53I3(PIG-3)*, and *BBC3(PUMA)*, was evaluated by qRT-PCR, and showed that combination treatments induced more transcripts than MDM2 inhibitors alone, particularly after 24 h treatment (Figure 2B and Figure 3B). No significant increase of *TP53* mRNA was found, so it was reasonable to hypothesize that trametinib modulates post-translational modification of p53 in cells after concurrent treatment with MDM2 inhibitors. To evaluate the changes in cell cycle distribution and apoptosis after treatments with either MDM2 inhibitors or trametinib, alone or in combination, fluorescence-activated cell sorting (FACS) and caspase 3/7 assay were performed. FACS showed the combination treatment induced more cell cycle arrest than single agent treatment, evidenced by decreases in S phase fractions and increases in either G1 or G2 phases (Figure 2C,D and Figure 3C). Furthermore, increased sub-G1 signals by FACS and increased caspase 3/7 activity were observed for A375 treated with combinations than for signal agent treatment, indicating more apoptosis was induced by the combination treatments (Figure 2E,F). This was compatible with increased p53 target transcripts (Figure 2B). Consistent with our previous report [9], there was no significant induction of apoptotic biomarkers, such as sub-G1 signals on FACS analysis or caspase 3/7 activity, in the WM35 cell line (Figure 3D,E).

### 2.3. The Combination Effects for Trametinib and MDM2 Inhibitors in Paired WM35/WM35-R Cell Lines

The combination effects were also explored in WM35-R [9], a p53 mutated (p53^MUT^, Gly334Val) cell line, selected from WM35 (Figure 3). Although the WM35-R cells remained responsive to trametinib (1 nM), there were no significant differences with and without the addition of RG7388 (0.2 µM) on protein responses (Figure 3A), transcripts (Figure 3B), or cell cycle distribution (Figure 3C,D), and only the effects of trametinib could be found in WM35-R treated with combination of trametinib and RG7388, indicating RG7388 played no role in the response of WM35-R, suggesting that the effects of combination seen in the p53^WT^ WM35 and A375 are p53 dependent.

### 2.4. DUSP6 Regulation by pERK Rather Than p53

**Figure 4:**
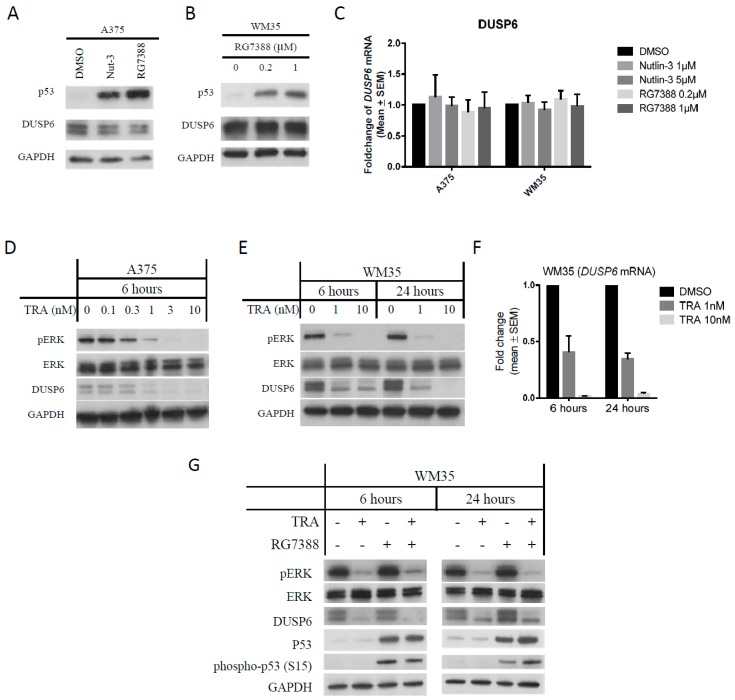
DUSP6 was regulated by pERK and not by p53. (**A**) Immunoblotting of A375 treated with nutlin-3 (5 µM) and RG7388 (1 µM) for 6 h. (**B**) Immunoblotting of WM35 treated with RG7388 (0.2 and 1 µM) for 6 h. (**C**) *DUSP6* mRNA in A375 and WM35 treated with the indicated concentrations of nutlin-3 and RG7388 for 6 h, measured by qRT-PCR. (**D**,**E**) Immunoblotting of A375 (**D**) and WM35 (**E**) treated with trametinib for the indicated concentrations and times. (**F**) Changes of DUSP6 mRNA by qRT-PCR in WM35 treated with trametinib. (**G**) Immunoblotting of WM35 treated with trametinib (1 nM) and/or RG7388 (0.2 µM) for 6 and 24 h. TRA, trametinib.

DUSP6, a phosphatase which acts on pERK, was also reported to be regulated by p53 and MAPK activity. Therefore, DUSP6 is a likely key candidate protein for involvement in connecting p53 and MAPK pathways, and could be important in the mechanism of synergy seen for combination treatment with MDM2 and MEK inhibitors. To examine whether DUSP6 is indeed regulated by p53 or pERK, A375 and WM35 were treated with MDM2 inhibitors or trametinib separately. There were no significant changes of DUSP6 protein and mRNA in either cell line treated with nutlin-3 or RG7388 (Figure 4A–C). However, significant dose-dependent suppression of DUSP6 was found after trametinib treatment (Figure 4D–F) and the decrease of DUSP6 occurred at an early time point of 2–4 h following pERK suppression (Supplementary Figure S3A). DUSP6 suppression was also found in A375 treated with the BRAF inhibitor, vemurafenib, offering additional evidence to support the observation that inhibition of MAPK activity suppresses DUSP6 (Supplementary Figure S3B). Therefore, we conclude that DUSP6 is regulated by MAPK activity but not by p53, at least in A375 and WM35 melanoma cells.

In addition, immunoblotting of WM35, following treatment with a combination of RG7388 and trametinib for 6 and 24 h, shows DUSP6 suppression to be associated with reduced phosphorylation of pERK (Figure 4G). Phospho-p53 (S15) was probed, as serine 15 is an important key residue in the post-translational modification of p53 [26]. The combination treatment with MEK and RG7388 inhibitors induced more phospho-p53 after 24 h than RG7388 alone, consistent with the hypothesis that DUSP6 suppression increases p53 phosphorylation. Similar results were found for A375 treated with trametinib and HDM201 (Supplementary Figure S4). Even though p53 phosphorylation status was increased in association with downregulation of DUSP6, it was not clear whether this is a direct or indirect effect of DUSP6 on p53 phosphorylation. It has been reported that phospho-ATM increases when DUSP6 is suppressed [27]. Since ATM phosphorylates p53, we further hypothesized that this may represent an indirect route for DUSP6 to regulate p53 phosphorylation via ATM.

### 2.5. DUSP6 Suppression by siRNA Promotes the Effect of MDM2 Inhibitors through ATM-Mediated p53 Phosphorylation

**Figure 5:**
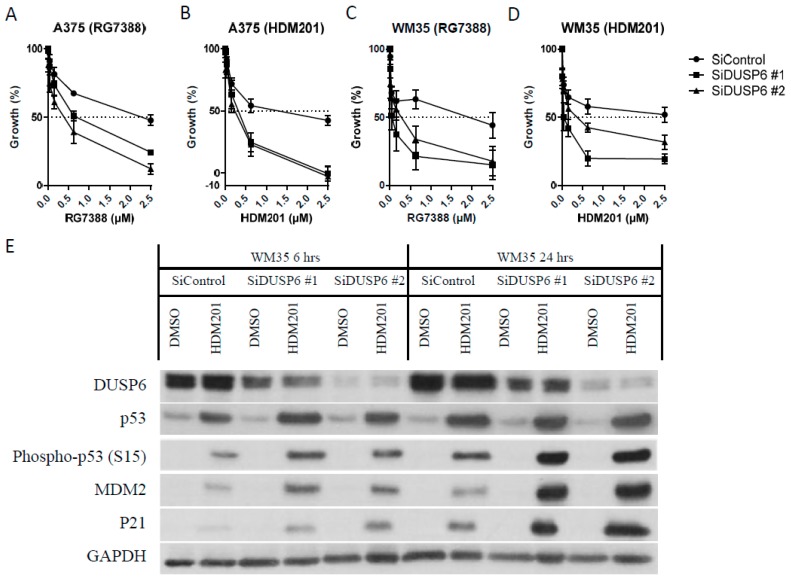

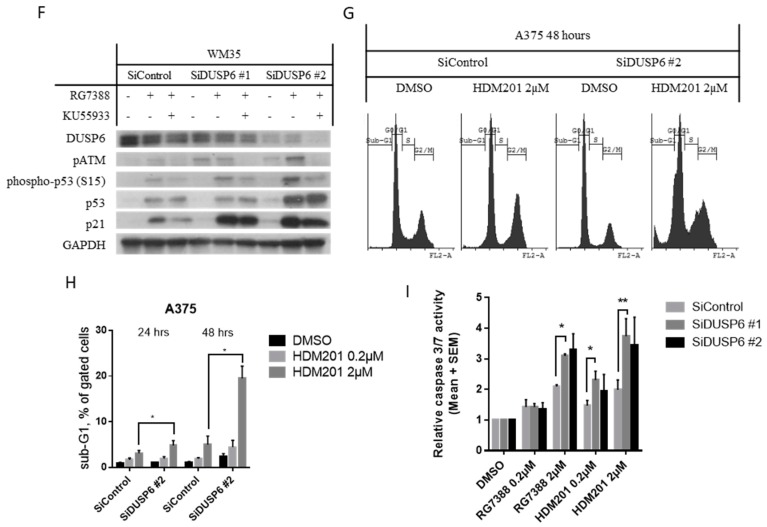
DUSP6 suppression by siRNA promotes the anticancer effect of MDM2 inhibitors through ATM-mediated p53 phosphorylation. (**A**–**D**) Growth inhibition of A375 (**A**,**B**) and WM35 (**C**,**D**) after 24-h treatment with siRNA against DUSP6 followed by 48-h RG7388 (**A**,**C**) or HDM201 (**B**,**D**) treatment. (**E**) Immunoblotting of WM35 after siRNA knockdown of DUSP6 for 24 h followed by treatment with 1 µM HDM201 for 6 and 24 h. (**F**) Immunoblotting of WM35 after 24-h DUSP6 siRNA knockdown, followed by 200nM RG7388 ± 10 µM KU55933 24-h treatment. (**G**,**H**) Flow cytometry of A375 treated with DUSP6 siRNA followed by HDM201 addition and presented as a histogram (**G**) and % of sub-G1 signals (**H**). (**I**) Caspase 3/7 assay of A375 after 24-h DUSP6 siRNA followed by RG7388 or HDM201 treatment with the indicated doses. hrs, hours; *, *p* < 0.05.

To investigate the causal link between DUSP6 suppression by trametinib and increased p53 phosphorylation and its activity, transient knockdown of DUSP6 by two alternative siRNA was performed. Both A375 and WM35 were transfected with 40 nM *DUSP6* siRNA for 24 h followed by treatment with MDM2 inhibitors, RG7388 or HDM201, for the indicated times. DUSP6 suppression by two alternative siRNA made both A375 and WM35 cell lines more sensitive to either RG7388 or HDM201 (Figure 5A–D). Immunoblotting of WM35 after siRNA-mediated DUSP6 knockdown followed by HDM201 treatment for 6 and 24 h demonstrated that higher levels of p53 phosphorylation (Ser15) and more induction of p21 and MDM2 occurred after DUSP6 suppression (Figure 5E). Similar results were found with A375 after siRNA knockdown and HDM201 treatment (Supplementary Figure S5A). Interestingly, pERK, a reported substrate of DUSP6, did not increase appreciably after DUSP6 suppression, possibly because of compensation by phosphatase activity from other DUSP family members [27], and/or pERK may be saturated by upstream kinases activated by the BRAF mutation.

The ATM inhibitor KU55933 was used to evaluate whether the DUSP6 regulation of phospho-p53 is ATM dependent. Immunoblotting of WM35 (Figure 5F) and A375 (Supplementary Figure S5B) treated with RG7388 after siRNA-mediated DUSP6 suppression showed the increased phospho-ATM (S1981) and phospho-p53 were reversed by the KU55933 ATM inhibitor. This indicates that DUSP6 inhibition by siRNA promotes p53 phosphorylation mainly through ATM, rather than by a direct effect on p53.

Significant increases in apoptosis induced by HDM201 after siRNA-mediated DUSP6 knockdown were also evident from sub-G1 signals by FACS analysis (Figure 5G,H, Supplementary Figures S6 and S7) and caspase 3/7 activity (Figure 5I). These observations reveal DUSP6 to be a negative regulator of p53 and, hence, a potential therapeutic target for potentiating the effect of MDM2 inhibitors in cutaneous melanoma.

### 2.6. DUSP6 Enzymatic Inhibitor (BCI) at a Dose without Cytotoxicity Promotes the Anticancer Effect of MDM2 Inhibitors through p53 Phosphorylation

**Figure 6:**
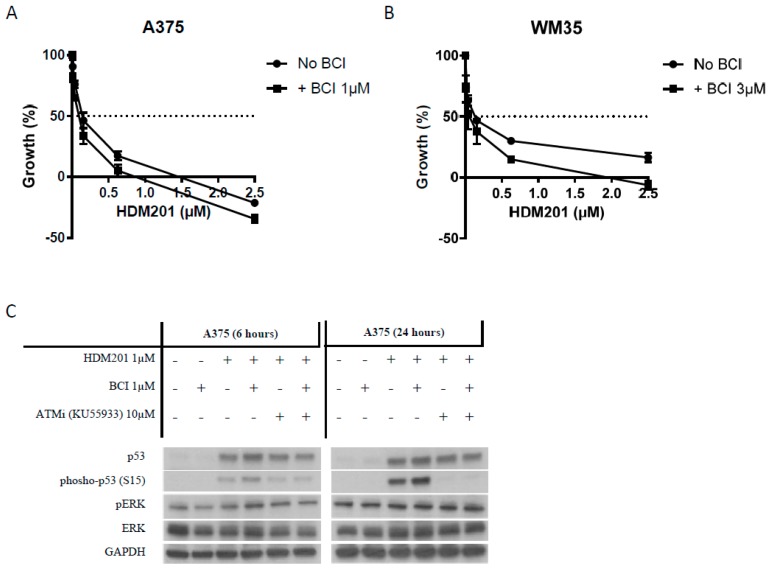
DUSP6 suppression by inhibitor (BCI) promotes the anticancer effect of MDM2 inhibitors through p53 phosphorylation. (**A**,**B**) Dose-dependent growth inhibition of A375 and WM35 treated with HDM201 ± BCI for 72 h. (**C**) Immunoblotting of A375 treated with HDM201 ± BCI ± KU55933 for 6 and 24 h.

To further explore the role and potential targeting of DUSP6-mediated negative regulatory signaling from the MAPkinase pathway to p53 in melanoma cells, we used a selective small molecule inhibitor of DUSP6, 2-benzylidene-3-(cyclohexylamino)-1-indanone hydrochloride (BCI) which inhibits DUSP6 allosterically [28,29]. The GI_50_ values of BCI for A375 and WM35 were 1.38 + 0.24 µM and 3.17 ± 0.21 µM respectively (Supplementary Figure S8). Since BCI was reported to show whole organism toxicity at concentrations more than 5 µM [30], BCI concentrations lower than the GI_50_ (1 µM for A375, 3 µM for WM35), with minimal effect on cell proliferation, were used for combination treatment with HDM201 and showed potentiation of HDM201 treatment (Figure 6A,B), which was consistent with the results of siRNA-mediated DUSP6 suppression. To examine whether the effects of potentiation are due to ATM-dependent p53 phosphorylation, immunoblotting of A375 treated with 1 µM HDM201 ± 1 µM BCI ± ATM inhibitor for 6, 24 h was performed (Figure 6C). BCI alone did not show any effects on p53 and its phosphorylation (Ser15), whereas increased p53 phosphorylation at Ser15 was found after HDM201 and BCI combination treatment compared with HDM201 alone. Furthermore, the increased phosphorylation was completely reversed by the KU55933 ATM inhibitor, indicating DUSP6 inhibition by BCI promotes p53 phosphorylation mainly through ATM, rather than a direct effect on p53.

## 3. Discussion

**Figure 7:**
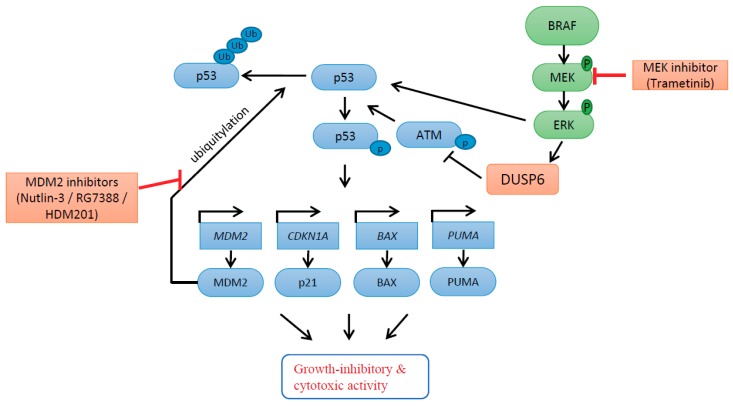
Proposed model involving DUSP6 for response to MEK inhibitor, trametinib, in combination with MDM2 inhibitors. DUSP6 is regulated by ERK rather than p53. Downregulation of DUSP6 by MEK inhibition with trametinib results in loss of DUSP6 phosphatase activity on ATM and consequent increased ATM-mediated phosphorylation of p53 after p53 release by MDM2 inhibitors (nutlin-3/RG7388/HDM201) in BRAF^V600E^/p53^WT^ cutaneous melanoma cells. These changes, leading to increased p53 phosphorylation, promote the growth-inhibitory and cytotoxic activity of p53 by increasing p53-dependent transcriptional activity.

In the current study, combined targeting of the MAPK and p53 pathways by trametinib and MDM2 inhibitors was found to be synergistic and to involve DUSP6 suppression by trametinib, followed by increased p53 phosphorylation. DUSP6 was shown to be regulated by ERK, rather than p53, and downregulation of DUSP6 by trametinib treatment resulted in reduced phosphatase activity on ATM and a consequent increase in p53 (Ser15) phosphorylation, after p53 activation by combined treatment with MDM2 inhibitors (nutlin-3/RG7388/HDM201) in BRAF^V600E^/p53^WT^ cutaneous melanoma cells. These changes promoted the growth-inhibitory and cytotoxic activity of p53 by increasing p53-dependent transcriptional activity (Figure 7). DUSP6 suppression by both siRNA knockdown of expression and enzymatic inhibition (BCI) showed similar enhancement of MDM2 inhibitor activity.

The effects of DUSP6 inhibition by trametinib, in the current study, were consistent with knockdown of DUSP6 expression by siRNA and direct enzymatic inhibition with BCI. All showed similar trends when combined with MDM2 inhibitors, except the time scales differed. The responses were evaluated at two different time points, 6 and 24 h. The early effects of enhanced p53 phosphorylation and activity were found after 6-h treatment with MDM2 inhibitors when DUSP6 was suppressed by siRNA or BCI (Figure 4E and Figure 6C), but not at this early time point, when DUSP6 was suppressed by trametinib (Figure 4G and Supplementary Figure S4). By contrast, the phosphorylation of p53 and induction of its transcriptional targets were particularly evident after 24-h combined trametinib and MDM2 inhibitor treatments. This may be due to the difference between trametinib and siRNA/BCI suppression of DUSP6, in that trametinib additionally suppresses pERK, which has been reported to be a direct kinase of p53, for instance, phosphorylating serine 15 in response to UV radiation [31]. Therefore, a balance between pERK and DUSP6 plausibly determines the p53 phosphorylation status. In addition, DUSP6 is not the only phosphatase which works on ATM/p53. Other phosphatases, such as WIP1, a direct transcriptional target of p53, can also contribute to negative regulation of p53 phosphorylation, although this was ruled out as a contributory factor in the mechanism of synergy between trametinib and MDM2 inhibitors in this study (Supplementary Figure S4).

Our observation of synergy is supported by a previous limited study using nutlin-3 (early tool MDM2 inhibitor) and two MEK inhibitors, U0126 (used as a research tool compound only) and AZD6244 (under clinical trials) [32]. However, no advanced mechanistic experiments and explanations were addressed in the previous study. Some possible explanations were discussed [33], and one plausible explanation is that MEK inhibition by U0126 initiated downregulation of the *MDM2* mRNA and protein, followed by increased p53 activity [34]. However, such a pattern was not found in our study (Figure 2A,B and Figure 3A,B). We investigated the combination with trametinib, a clinically approved compound, and MDM2 inhibitors, nutlin-3/RG7388/HDM201 (the latter two are in clinical trials) in melanoma cell lines, which has direct translational implications to the clinical setting. In addition, our detailed study revealed a novel mechanism, which involves DUSP6 and p53 phosphorylation mediated by ATM, offering another potential therapeutic strategy for enhancing non-genotoxic p53 reactivation by MDM2 inhibitors.

Combination and dose reduction index values were used to evaluate the synergism and dose reduction potential of these combinations [25]. Favorable dose reductions (DRI > 1.0) (Table 1) for the combination of trametinib and MDM2 inhibitors implies this combination can reduce the individual drug dose to reach the same therapeutic effect as higher individual doses, leading to decreased side effects compared with the single agents, since they have differing dose-limiting toxicities. In the case of MDM2 inhibitors, the dose-limiting toxicities are hematological (neutropenia and thrombocytopenia [17,35], whereas for trametinib, the dose-limiting toxic effects include rash, diarrhea, and central serous retinopathy [36].

In addition to providing a mechanistic insight into the synergistic effects of trametinib and MDM2 inhibitors, DUSP6, as a negative regulator of ATM/p53, was identified as a potential therapeutic target for enhancing the effect of p53 activators. DUSP6 expression in cutaneous melanoma is the highest among all cancer cell lines and the second highest among cancer tissue types (Supplementary Figure S1), suggesting DUSP6 plays a particularly important role in cutaneous melanoma. Consistent with previous studies reporting DUSP6 regulation of the DNA damage response pathway, in which DUSP6 suppression was associated with elevated levels of phosphorylated H2AX, ATM, and CHEK2, [27] we found that DUSP6 regulated p53 phosphorylation via ATM, even when p53 is released and activated by MDM2 inhibitors in a non-genotoxic manner.

We failed to find obvious changes of DUSP6 expression after activation of p53 by MDM2 inhibitors in melanoma cell lines, despite clear induction of p53 and a previous suggestion of DUSP6 as a p53 direct transcriptional target [18]. This previous study reported the identification of two putative p53 binding half-sites in the DUSP6 promoter region and chromatin immunoprecipitation (ChIP) analysis to support direct p53 binding to the DUSP6 promoter [18]. However, global analysis of p53 transcription-factor binding sites by ChIP with the paired-end ditag (PET) sequencing strategy did not identify DUSP6 as a transcriptional target [37]. Furthermore, none of the 16 datasets extracted from 13 genome-wide studies identify DUSP6 as a transcriptional target of p53 [38]. These collective lines of evidence did not support DUSP6 being a p53 direct transcriptional target, and is more consistent with the lack of induced DUSP6 expression by MDM2 inhibitors, alone, in our study.

In this study, the indirect regulation of p53 phosphorylation by DUSP6 via ATM is evidenced by the results of DUSP6 suppression using three different methods (MEK inhibitor, DUSP6 siRNA, and DUSP6 inhibitor). Phospho-p53 (S15) was induced more after MDM2 treatment under DUSP6 suppression than without DUSP6 suppression. In addition, we also probed phospho-ATM (S1981), which showed induction under DUSP6 suppression. Furthermore, the ATM inhibitor, KU55933, was used to reverse the increases in phospho-p53 and phospho-ATM, which demonstrated that these increases in phospho-p53 and phospho-ATM were ATM-dependent.

## 4. Materials and Methods

### 4.1. Cell Lines and Reagents

A375 (p53^WT^), and paired WM35 (p53^WT^)/WM35-R (p53^MUT^) cutaneous melanoma cell lines [9] were used in the study and were routinely cultured using Dulbecco’s modified Eagle’s medium (DMEM) medium, which was supplemented with 10% (v/v) fetal calf serum. All the cell lines were authenticated by serial tandem repeat (STR) profiling (NewGene, Newcastle, UK). Nutlin-3 was purchased from NewChem (Newcastle, UK), and RG7388 and HDM201 were sourced by custom synthesis (Astex Pharmaceuticals, Cambridge, UK). Trametinib and vemurafenib were purchased from Cambridge Bioscience (Cambridge, UK). All compounds were initially dissolved in dimethyl sulfoxide (DMSO) (Sigma-Aldrich, Gillingham, UK) and used at a final concentration of 0.5% DMSO, optimized to give minimal cytotoxic effects on cells, and 0.5% DMSO solvent controls were included in all experiments.

### 4.2. Growth Inhibition and Clonogenic Survival Assays

For growth inhibition studies, cells were seeded in 96-well plates overnight, and treated with indicated drugs for 72 h. The cells were fixed using Carnoy’s fixative followed by sulforhodamine B (SRB) staining and quantification, as previously described [9,39]. For clonogenic survival, cells were seeded in 6-well plates overnight, and treated with indicated drugs for 72 h. Fresh medium was replaced, and the cells were fixed and stained with crystal violet when visible colonies formed [40].

### 4.3. Combination Treatment

The effect of MDM2 inhibitors, nutlin-3/RG7388/HDM201, in combination with trametinib, was investigated for two BRAF^V600E^ and p53^WT^ melanoma cancer cell lines using median-effect analysis [25]. The growth inhibition of these cell lines was determined after 72 h exposure to nutlin-3, RG7388, HDM201, or trametinib, and in combination at five concentrations and a constant ratio, with a range covering their respective GI_50_ concentrations.

To determine whether the differences in response for growth inhibition were synergistic, additive or antagonistic, the data were analyzed using median-effect analysis, and CI and DRI values were calculated using CalcuSyn (Biosoft, Ferguson, MO, USA). CI values for each constant ratio combination and at effect levels of ED_50_, ED_75_, ED_90_, and ED_95_ were computed.

### 4.4. Immunoblotting

Cells lysates were harvested by 2% SDS lysis buffer, heated and sonicated. The protein concentrations of the cell lysates were estimated using a Pierce^®^ BCA Protein Assay kit. The detailed protocol used for immunoblotting can be found in our previous report [9]. The primary and secondary antibodies used in the current study were listed as follows: p53 (DO-7) (#M7001, Dako, Glostrup, Denmark), MDM2 (Ab-1) (#OP46, Merck Millipore, Watford, UK), p21^WAF1^ (EA10) (#OP64, Calbiochem (Millipore, Watford, Herts, UK), p-ERK (E-4) (#sc-7383, Santa Cruz, Dallas, TX, USA), ERK (K-23) (#sc-94, Santa Cruz), GAPDH (14C10) (#2118, Cell Signaling Technology, Danvers, MA, USA), WIP1 (F-10) (#sc-376257, Santa Cruz Biotechnology), phospho-p53 (S15) (ab1431, Abcam, Cambridge, UK), phospho-ATM(S1981) (#AF1655, R&D systems, Abingdon, UK), actin (#A4700, Sigma-Aldrich), secondary goat anti-mouse/rabbit horseradish peroxidase (HRP)-conjugated antibodies (#P0447/P0448, Dako).

### 4.5. RNA Extraction and qRT-PCR

Cells were treated with the indicated drugs for the indicated times and then were collected for RNA extraction, reverse transcription, and qRT-PCR. The detailed protocol and analysis method was reported in our previous publication [9].

The primers used in current study: *MDM2*(NM_002392): F—AGTAGCAGTGAATCTACAGGGA, R—CTGATCCAACCAATCACCTGAAT; *CDKN1A*(NM_000389): F—TGTCCGTCAGAACCCATGC, R—AAAGTCGAAGTTCCATCGCTC; *BBC3(PUMA)*(NM_001127240): F—ACCTCAACGCACAGTACGA, R—CTGGGTAAGGGCAGGAGTC; *BAX*(NM_001291428): F—CCCGAGAGGTCTTTTTCCGAG, R—CCAGCCCATGATGGTTCTGAT; *TP53I3(PIG-3)*(NM_004881): F—AGCGAGGAAGTCTGATCACC, R—CGTGGAGAAGTGAGGCAGAA; *TP53*(NM_000546) F—CAGCACATGACGGAGGTTGT, R—TCATCCAAATACTCCACACGC; *GAPDH* (NM_002046): F—CAATGACCCCTTCATTGACC, R—GATCTCGCTCCTGGAAGAT.

### 4.6. Fluorescence-Activated Cell Sorting (FACS)

Cells were treated with the indicated drugs for the indicated times and all floating and adhered cells were collected and fixed by 70% cold ethanol for staining by propidium iodide (Sigma-Aldrich, Dorset, UK). Detailed materials can be found in our previous report [9].

### 4.7. Caspase 3/7 Activity Assay

Melanoma cells were seeded in white 96-well plates (Greiner Bio-One, Stonehouse, Glos, UK) and treated after 24 h. Caspase-3/7 enzymatic activities were measured using a FLUOstar Omega plate reader (BMG Labtech, Aylesbury, UK) after adding a 1:1 ratio of CaspaseGlo 3/7 reagent (Promega, Southampton, UK) to growth media and incubating for 30 min. All values were expressed as a ratio of signal relative to solvent control.

### 4.8. SiRNAs and Transfection

A final concentration of 40nM siRNA duplex (Eurogentec, Hampshire, UK) against *DUSP6* and control noncoding sequence was used for transfection with Lipofectamine 2000 (Thermo Fisher Scientific) in Opti-MEM Reduced Serum Media (Thermo Fisher Scientific). The sequences were designed as follows: Control SiRNA (SiControl), sense: 5′-GCGCGCUUUGUAGGAUUCGdTdT-3′, antisense: 5′-CGAAUCCUACAAAGCGCGCdTdT-3′; two alternative *DUSP6* targeted siRNA (SiDUSP6, NM_001946), SiDUSP6 #1, sense: 5′- UAGCACGGAGUCCGAAUUAAU dTdT-3′, antisense: 5′-AUUAAUUCGGACUCCGUGCUA dTdT-3′, SiDUSP6 #2, sense: 5′-UACGGACACUAUUAUCACUAAdTdT-3′, antisense: 5′-UUAGUGAUAAUAGUGUCCGUAdTdT-3′.

### 4.9. Statistical Analysis

Data were presented as mean ± standard error of the mean (SEM) unless otherwise stated. Statistical tests were carried out using GraphPad Prism 6 software and all *p*-values represent paired *t*-tests of at least three independent repeats. A *p*-value less than 0.05 was considered as statistically significant.

## 5. Conclusions

In summary, we report the novel finding that MDM2 inhibition effectively activates p53 function and synergizes with trametinib as a result of increased p53 phosphorylation via trametinib-induced reduction in DUSP6 expression. Furthermore, this involves an indirect ATM-dependent step. The lower DUSP6 levels induced by trametinib inhibition of MEK result in increased phosphorylated ATM levels which, in turn, results in increased ATM-dependent p53 phosphorylation (Figure 7). These results have direct clinical implications and indicate potential ways of improving the efficacy of current targeted treatment with MAPK pathway inhibitors in BRAF^V600E^, p53^WT^ melanoma.

## Figures and Tables

**Table 1:** Summary of dose reduction index (DRI) for the combination treatment in A375, WM35.

Cell Line	ED50		ED75		ED90	
	Nutlin-3	Trametinib	Nutlin-3	Trametinib	Nutlin-3	Trametinib
**A375**	1.52 ± 0.05	3.43 ± 0.86	1.63 ± 0.05	3.49 ± 0.79	1.75 ± 0.07	3.58 ± 0.74
**WM35**	1.66 ± 0.73	4.15 ± 1.19	7.34 ± 1.45	3.93 ± 1.12	62.1 ± 14.3	3.72 ± 1.05
	**RG7388**	**Trametinib**	**RG7388**	**Trametinib**	**RG7388**	**Trametinib**
**A375**	4.84 ± 0.75	3.30 ± 0.77	18.3 ± 5.94	3.31 ± 0.71	42.6 ± 20.6	3.33 ± 0.66
**WM35**	0.87 ± 0.19	2.85 ± 1.12	9.24 ± 4.57	3.70 ± 0.82	61.7 ± 2.37	9.93 ± 6.71
	**HDM201**	**Trametinib**	**HDM201**	**Trametinib**	**HDM201**	**Trametinib**
**A375**	2.44 ± 0.57	2.63 ± 0.48	2.97 ± 0.51	7.17 ± 2.27	3.84 ± 0.76	22.2 ± 10.1
**WM35**	3.23 ± 0.58	4.48 ± 0.86	2.30 ± 0.58	9.41 ± 2.06	2.41 ± 0.97	25.9 ± 9.57

ED, effective dose.

## Supplementary Materials

The following are available online at http://www.mdpi.com/2072-6694/11/1/3/s1, Figure S1: *DUSP6* mRNA expression in different cancer types, Figure S2: Clonogenic survival of A375 treated with trametinib, RG7388 and HDM201 for 72 h, Figure S3: Immunoblotting of A375 and WM35 treated with trametinib or vemurafenib, Figure S4: Immunoblotting of A375 cells treated with trametinib, HDM201, or combinations of the two compounds for 6 and 24 h, Figure S5: Immunoblotting of A375 after DUSP6 siRNA knockdown, followed by 200 nM RG7388 ± 10 µM KU55933, Figure S6: Cell cycle distribution (A–D) and Sub-G1 phase (E) of A375 cells after treatment with two siRNA against DUSP6 for 24 h, followed by HDM201 addition for 24 and 48 h, Figure S7: Cell cycle distribution (A–D) and Sub-G1 phase (E,F) of WM35 cells after treatment with two siRNA against DUSP6 for 24 h, followed by HDM201 addition for 24 and 48 h, Figure S8: Growth inhibition of A375 and WM35 cells treated with the BCI DUSP6 inhibitor.
