# Supplementary material for: ATM Dependent DUSP6 Modulation of p53 Involved in Synergistic Targeting of MAPK and p53 Pathways with Trametinib and MDM2 Inhibitors in Cutaneous Melanoma
Source: Cancers (Basel). 2018 Dec 20;11(1):3. doi: 10.3390/cancers11010003 (PMC6356368; doi:10.3390/cancers11010003)
Supplement: Supplementary file 1 [file cancers-11-00003-s001.pdf]

ATM Dependent DUSP6 Modulation of p53 Involved in Synergistic Targeting of MAPK and p53 Pathways with Trametinib and MDM2 Inhibitors in Cutaneous Melanoma

Chiao-En Wu, Tsin Shue Koay, Arman Esfandiari, Yi-Hsuan Ho, Penny Lovat and John Lunec

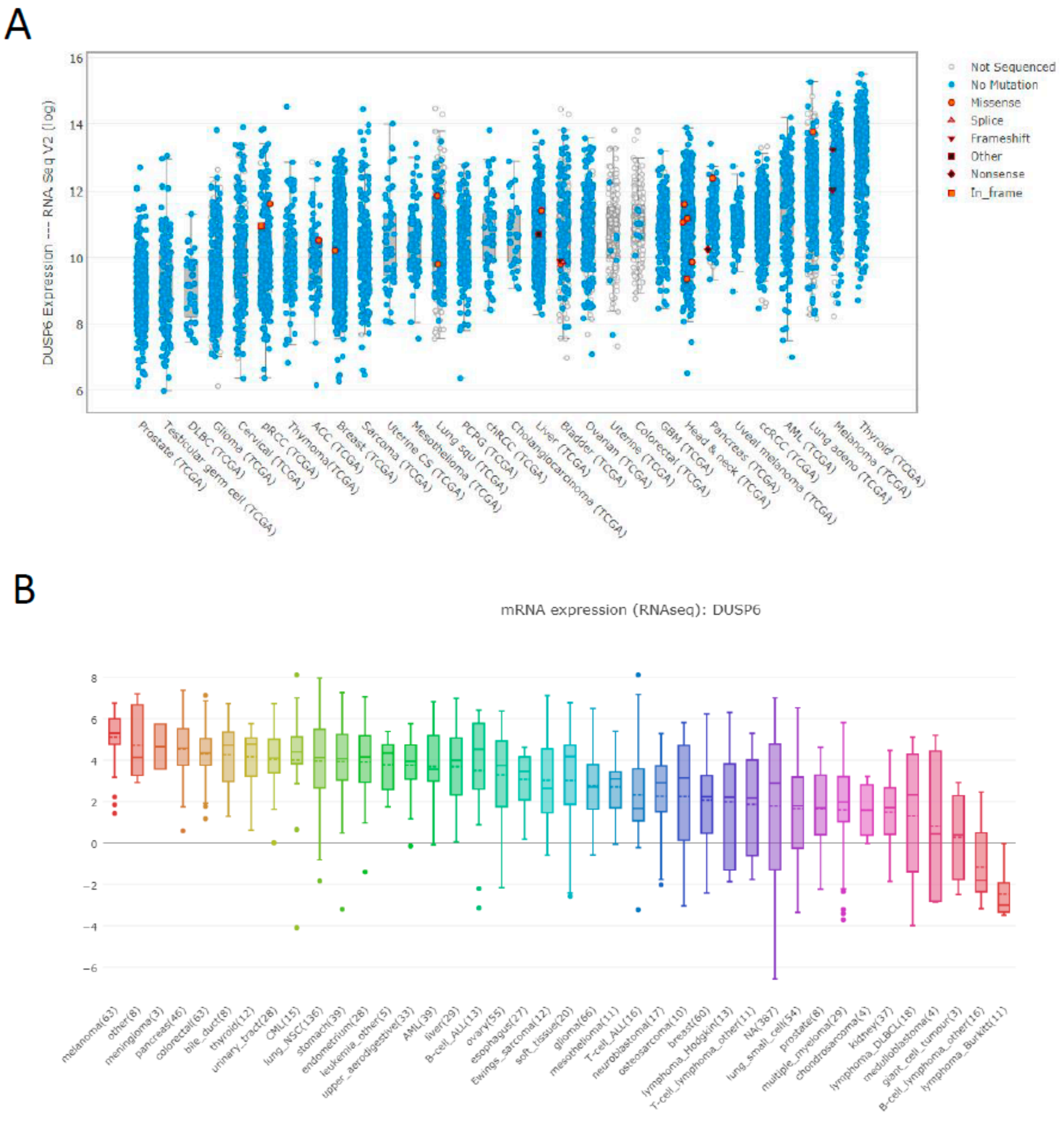

Figure S1. *DUSP6* mRNA expression in different cancer types. *DUSP6* mRNA expression in cBioPortal (**A**) and the Cancer Cell Line Encyclopaedia (CCLE) database (**B**).

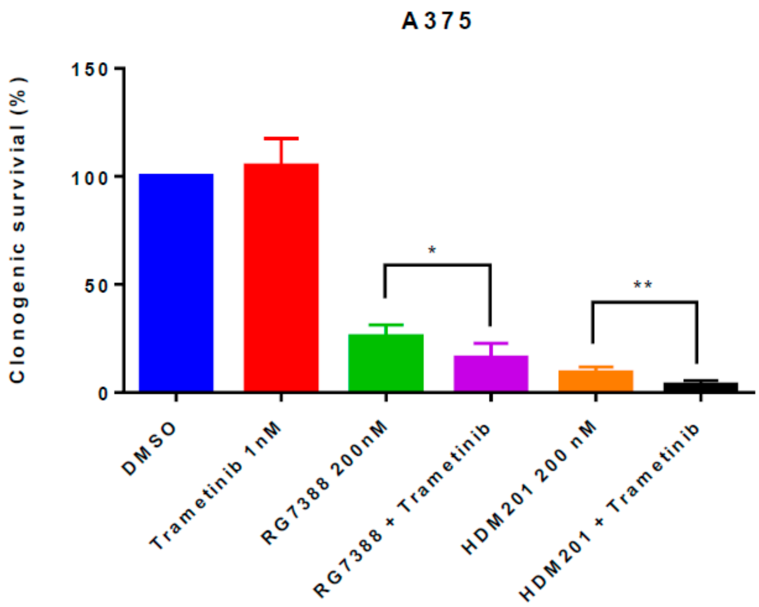

Figure S2. Clonogenic survival of A375 treated with trametinib, RG7388 and HDM201 for 72 hours. *, $p < 0.05$; **, $p < 0.01$

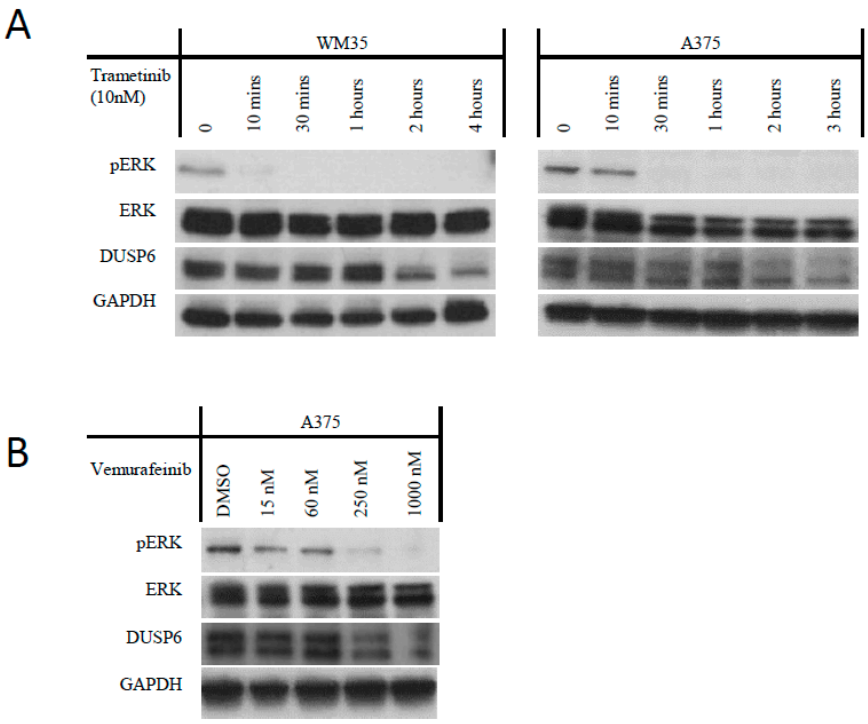

Figure S3. Immunoblotting of A375 and WM35 treated with trametinib or vemurafenib. (**A**) Immunoblotting of WM35 and A375 cells treated with 10nM trametinib for indicated times. (**B**) Immunoblotting of A375 cells treated with vemurafenib for 3 hours.

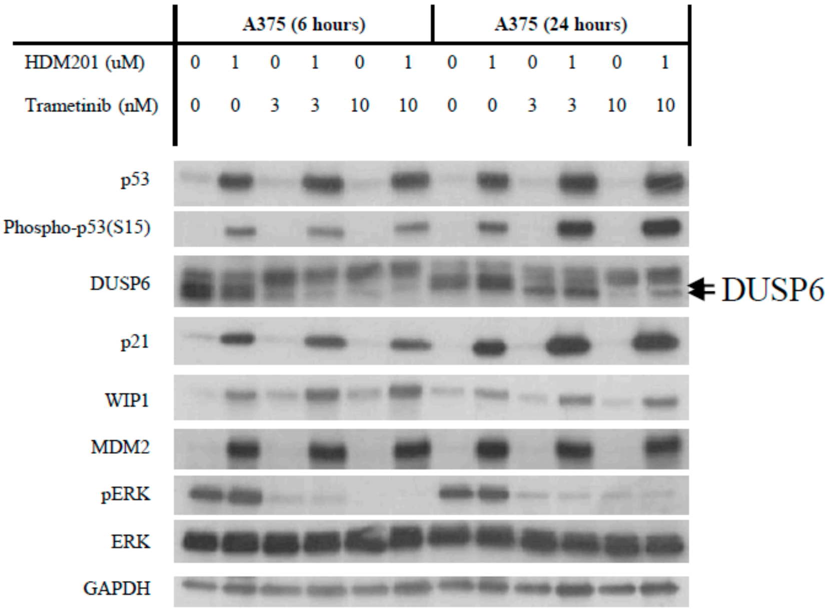

Figure S4. Immunoblotting of A375 cells treated with trametinib, HDM201, or combinations of the two compounds for 6 and 24 hours.

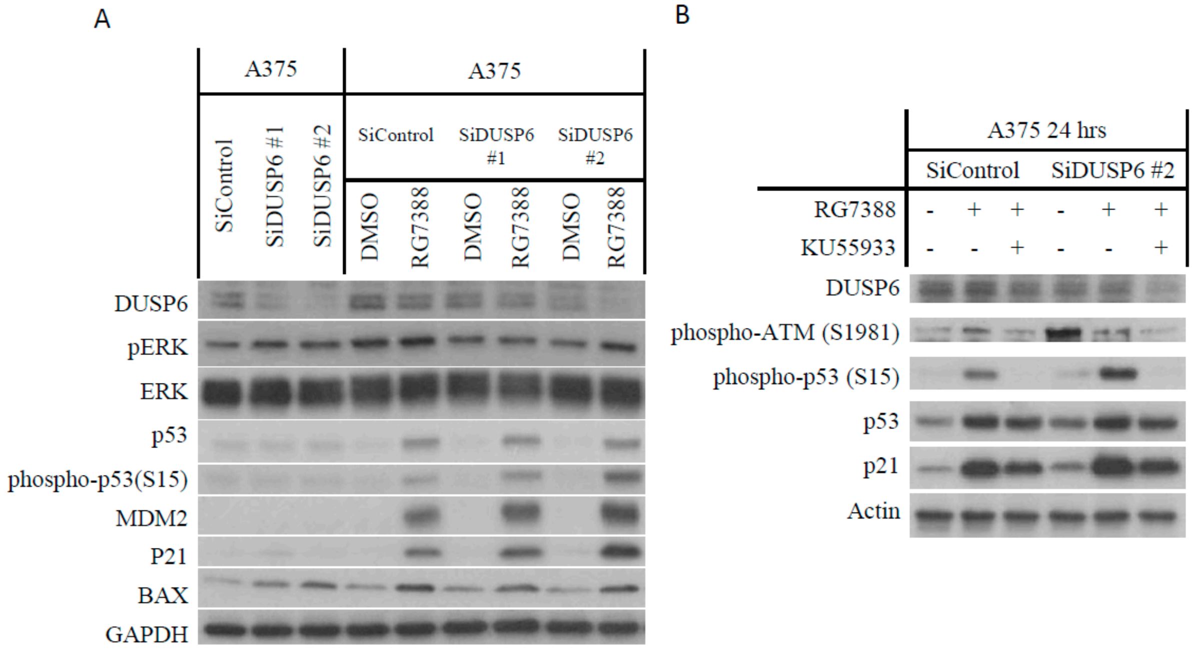

Figure S5. Immunoblotting of A375 after DUSP6 siRNA knockdown, followed by 200 nM RG7388 ± 10μM KU55933. (**A**) Immunoblotting of A375 after 24-hour DUSP6 siRNA knockdown, followed by 200nM RG7388 for 24 hours. (**B**) Immunoblotting of A375 after 24-hour DUSP6 siRNA knockdown, followed by 200nM RG7388 ± 10μM KU55933 24hr treatment. hrs, hours.

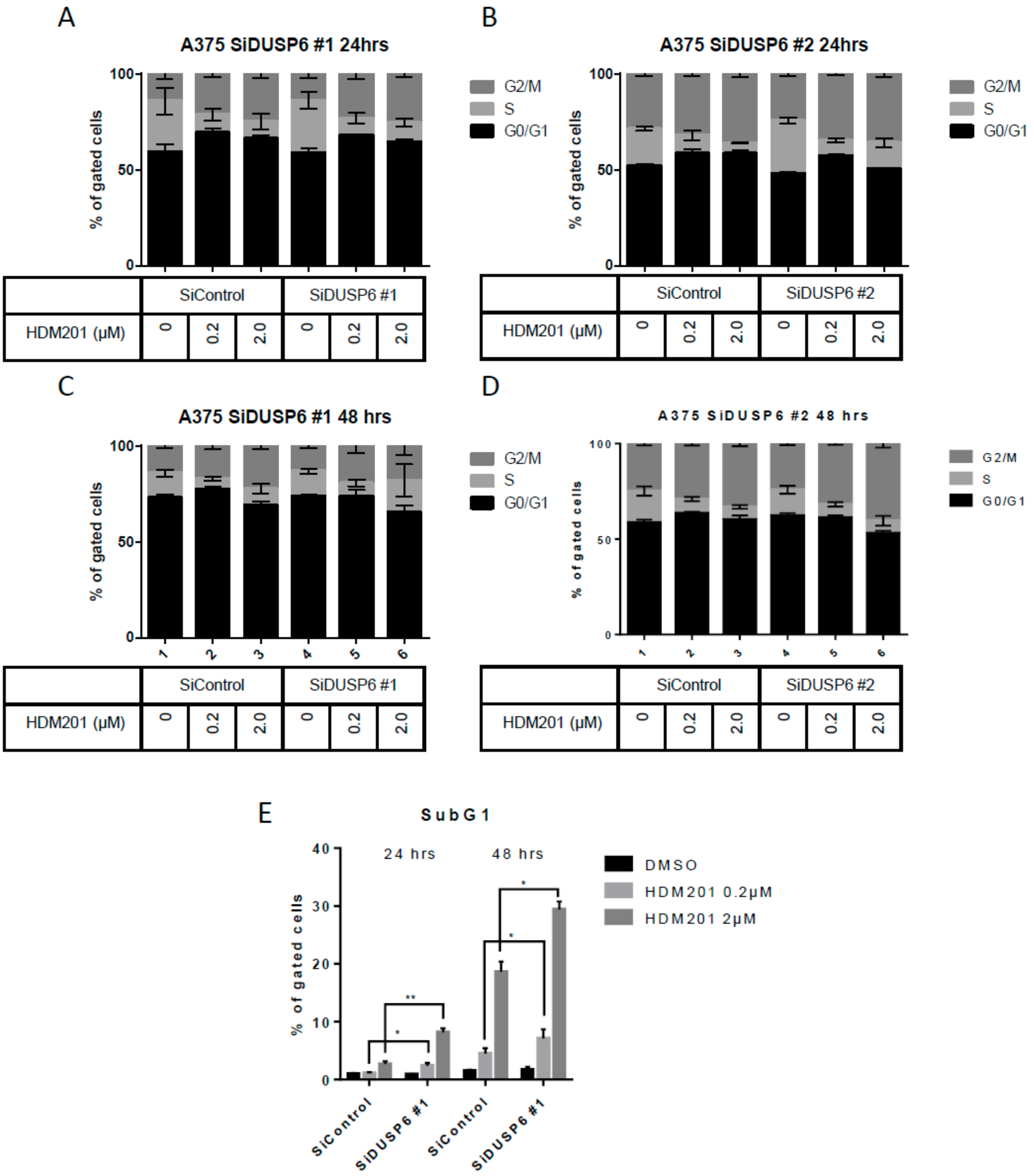

Figure S6. Cell cycle distribution (**A**–**D**) and Sub-G1 phase (**E**) of A375 cells after treatment with two siRNA against DUSP6 for 24 hours, followed by HDM201 addition for 24 and 48 hours. hrs, hours; *, $p < 0.05$; **, $p < 0.01$.

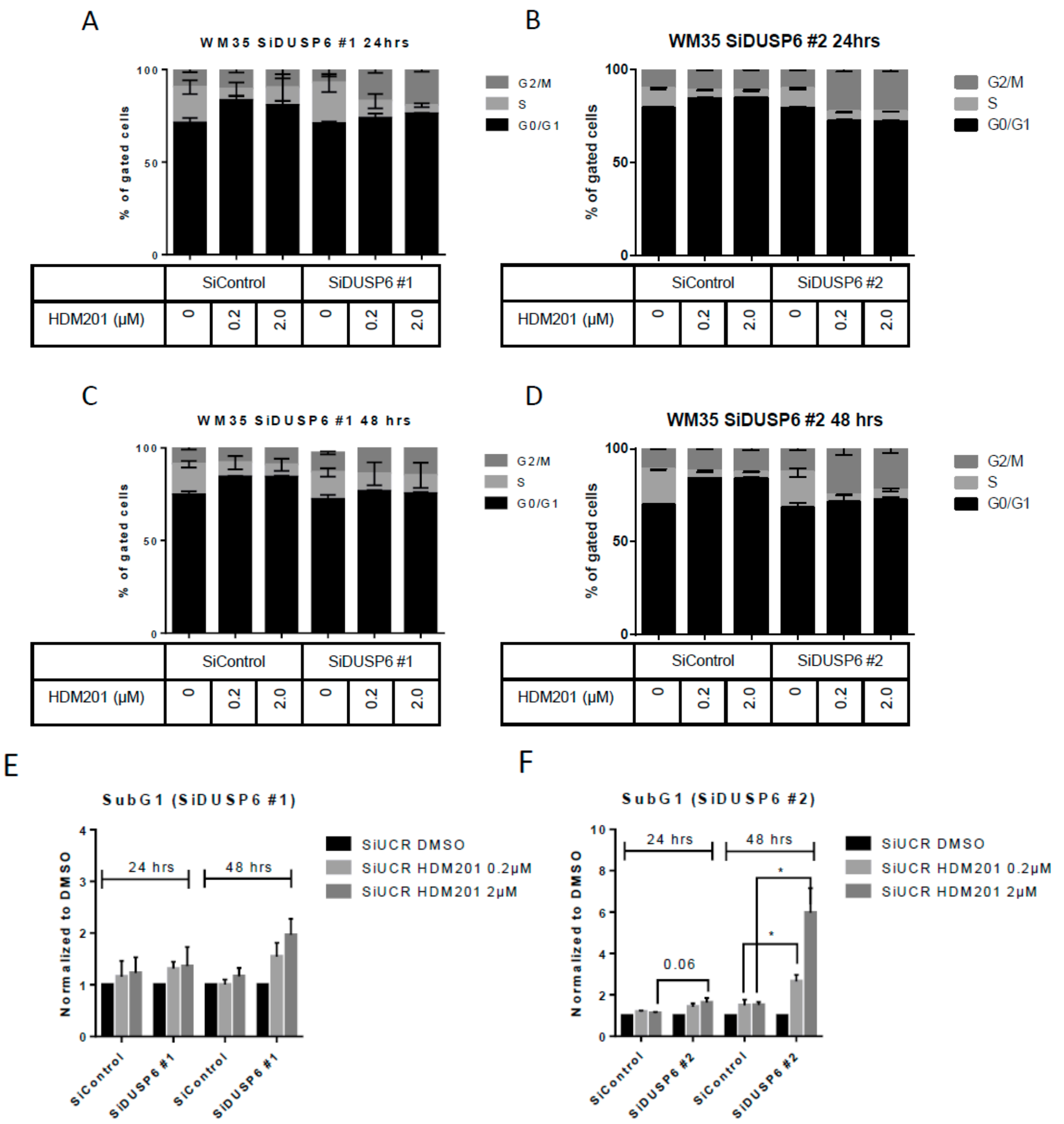

Figure S7. Cell cycle distribution (**A**–**D**) and Sub-G1 phase (**E**,**F**) of WM35 cells after treatment with two siRNA against DUSP6 for 24 hours, followed by HDM201 addition for 24 and 48 hours. hrs, hours; *, $p < 0.05$; **, $p < 0.01$.

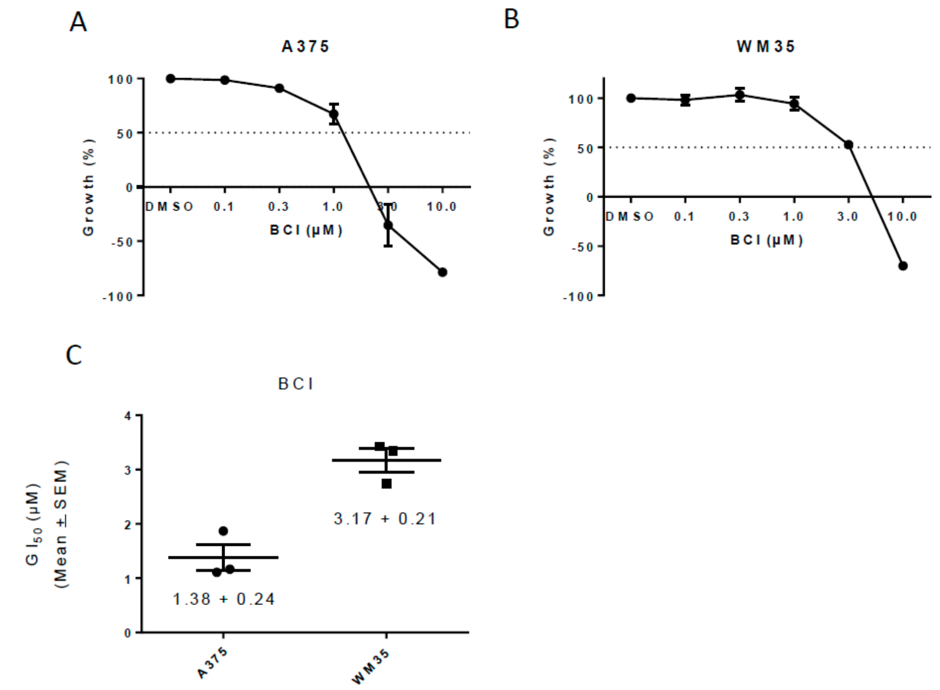

Figure S8. Growth inhibition of A375 and WM35 cells treated with the BCI DUSP6 inhibitor. (**A**,**B**) Growth inhibition of A375 and WM35 cells treated with the BCI for 72 hours. (**C**) Summary of GI_{50} values for BCI induced growth inhibition of A375 and WM35 cells.

© 2018 by the authors. Licensee MDPI, Basel, Switzerland. This article is an open access article distributed under the terms and conditions of the Creative Commons Attribution (CC BY) license (http://creativecommons.org/licenses/by/4.0/).
